# Science organisations and Coca-Cola’s ‘war’ with the public health community: insights from an internal industry document

**DOI:** 10.1136/jech-2017-210375

**Published:** 2018-03-14

**Authors:** Pepita Barlow, Paulo Serôdio, Gary Ruskin, Martin McKee, David Stuckler

**Affiliations:** 1 Department of Sociology, University of Oxford, Oxford, UK; 2 US Right to Know, Oakland, California, USA; 3 Department of Public Health and Policy, London School of Hygiene and Tropical Medicine, London, UK; 4 Department of Policy Analysis and Public Management, Bocconi University, Milan, Italy

**Keywords:** obesity, nutrition, health policy

## Abstract

Critics have long accused food and beverage companies of trying to exonerate their products from blame for obesity by funding organisations that highlight alternative causes. Yet, conclusions about the intentions of food and beverage companies in funding scientific organisations have been prevented by limited access to industry’s internal documents. Here we allow the words of Coca-Cola employees to speak about how the corporation intended to advance its interests by funding the Global Energy Balance Network (GEBN). The documents reveal that Coca-Cola funded and supported the GEBN because it would serve as a ‘weapon’ to ‘change the conversation’ about obesity amidst a ‘growing war between the public health community and private industry’. Despite its close links to the Coca-Cola company, the GEBN was to be portrayed as an ‘honest broker’ in this ‘war’. The GEBN’s message was to be promoted via an extensive advocacy campaign linking researchers, policy-makers, health professionals, journalists and the general public. Ultimately, these activities were intended to advance Coca-Cola’s corporate interests: as they note, their purpose was to ‘promote practices that are effective in terms of both policy and profit’. Coca-Cola’s proposal for establishing the GEBN corroborates concerns about food and beverage corporations’ involvement in scientific organisations and their similarities with Big Tobacco.

## Introduction

Food and beverage companies have long been accused of trying to exonerate their products from blame for increasing rates of obesity by implicating declining physical activity instead.^1 2^ In August 2015, these concerns reached a new audience when the New York Times revealed how Coca-Cola had spent $1.5 million to create the Global Energy Balance Network (GEBN) to disseminate messages about obesity focused on the role of ‘energy balance’.^3^ This portrayed obesity as about whether caloric intake was out of balance with exercise, rather than what or how much food and beverages people consume. Crucially, it was easier to achieve ‘energy balance’ with high levels of exercise and caloric intake.^4^

Commentators draw comparisons between organisations like the GEBN and tobacco industry-funded organisations, so-called ‘Merchants of Doubt’ who diverted attention away from secondhand smoke as a cause of disease by challenging research showing its risks and invoking other causes of observed associations.^3 5 6^ Food and beverage industry executives and the researchers they fund reject such comparisons, arguing that corporate funding does not mean that recipients advance corporate interests.^7 8^ While this view has been challenged, industry intentions have so far only been inferred from what they fund rather than established with certainty.^9–11^ There has been relatively little access to the industry’s internal documents, as was the case with Big Tobacco through legal challenges, where this definitively exposed the many ways that it promoted its business interests in scientific debates about tobacco—at the expense of public health.^12 13^

Here we allow the words of employees of a food and beverage corporation—Coca-Cola—to speak about how it intended to advance its interests by funding a scientific organisation. This unique source is Coca-Cola’s proposal to establish the GEBN, obtained in 2016 by US Right to Know, a consumer and public health group, through a state Freedom of Information request.^i^ The proposal was attached to an email sent by Rhona Appelbaum, former Chief Health and Science officer at Coca-Cola, to a small group of academics on 9 July 2014. The emails show how Coca-Cola intended to use the GEBN to: (i) reframe obesity as a matter of addressing ‘energy balance’; (ii) portray the GEBN as an ‘honest broker’ in the obesity debate; (iii) promote obesity reduction strategies that are commensurate with Coca-Cola’s interests via an extensive advocacy campaign.^14–16^

## Strategy 1: advance ‘energy balance’ as the right framework to deal with obesity

Coca-Cola’s proposal for establishing the GEBN shows how the company wanted to use the GEBN to ‘change the conversation’ about the causes of obesity. To reorient the debate, the GEBN was proposed to ‘advance ‘energy balance’ as the appropriate framework for addressing obesity’.

Coca-Cola’s proposal portrays the interests of public health as in conflict with their own. This is evident in the proposal from the argument that the science of ‘energy balance’ could be deployed as a ‘weapon’ in the ‘growing war between the public health community and private industry’ over obesity. Coca-Cola was concerned that the company was losing this battle. As the proposal states, the company had ‘failed to develop… an alternative to strategies being proposed’. In spreading the ‘energy balance’ message, the GEBN would help ‘to counter the voices touting extreme solutions to the obesity problem, for example, food is tobacco’. By referring to ‘extreme solutions’ or ‘unreasonable views’, Coca-Cola referred to government regulations to tax or ban foods that are considered unhealthy.

Importantly, the proposal stated that the GEBN should not aim to attack directly these ‘unreasonable views’. Instead, Coca-Cola sought to promote a narrative that could challenge the view that diet played a leading role in obesity: the GEBN would ‘play offence with alternative solutions’ rather than ‘defending the status quo’.

## Strategy 2: establish an ostensibly independent broker with assistance from scientists sympathetic to its goals

Coca-Cola’s GEBN proposal aimed to establish ‘a credible ‘honest broker’ in this battle who can be a reliable and trusted source for a balanced, science-based view’. However, this presupposes that such a broker would be fully independent of corporate interests. Indeed, Coca-Cola made a concerted effort at distancing itself from the GEBN to conceal its involvement. As Appelbaum wrote to GEBN academics Steven Blair and James Hill on 6 March 2014, “We need to be hands-off as the GEBN begins to take of. This is essential… A labor of love, but we need to make sure you are as independent as soon as possible.” Yet, as the documents show, the GEBN and its message were not independent, as Coca-Cola was promoting a scientific standpoint to academics and offering funding.

The documents also reveal Coca-Cola’s attempts at influencing the scientific community. First, the proposal states that the GEBN would ‘facilitate new thinking within the science of energy balance’. This would build on previous experience in ‘engaging experts… to frame problems differently’. The GEBN would develop white papers to ‘guide the field towards solutions to obesity based on the science of energy balance’. Second, the GEBN would serve ‘as a conduit to linking funding sources with innovative new research ideas’ and ‘the most influential researchers using an energy balance approach’. Finally, the GEBN would ‘empower them (the scholars) to promote this approach’ at ‘scientific societies and at scientific meetings’, while encouraging ‘ongoing submissions to scientific and consumer publications’.

All of these might be considered reasonable objectives had they emerged from a truly independent process but, as the proposal indicates, this was not the case.

## Strategy 3: convince policy-makers, journalists and the public that ‘energy balance’ is the right framework for addressing obesity

The proposal for establishing the GEBN placed a substantial emphasis on the GEBN’s wider communications strategy. This spanned a plethora of political activities that would form ‘a multiyear advocacy campaign’. And, ‘the consistent message from the GEBN’ across this campaign was that ‘an energy balance framework is the only framework that makes sense in addressing obesity’. The comment made in the proposal that the programme would operate in a manner ‘akin to a political campaign’ suggests that this was seen as somewhat different from the usual public engagement by researchers.

The proposal states that this ‘advocacy campaign’ would target five main groups. First, the GEBN would educate policy-makers about ‘why the energy balance framework is the right way to approach obesity’. The proposed education ‘tactics’ were ‘one-on-one meetings with policy makers’, ‘meetings focused on energy balance’ and ‘policy and white papers targeted toward policy-makers’. The GEBN would also attempt to access policy domains that might otherwise be inaccessible as they sought to ‘nominate GEBN scholars for key government panels’.

Second, Coca-Cola proposed that the GEBN would create a ‘programme for using the energy balance approach to teach healthcare professionals how to address obesity’. Third, the GEBN would expand its efforts in educating ‘health and wellness journalists’ and ‘national fitness and health bloggers’ about ‘energy balance’. This involved workshops, internships and ‘annual education conferences’. Ultimately, Coca-Cola aimed to establish the GEBN ‘as the place media goes to for a comment on any obesity issue’.

Fourth, Coca-Cola proposed that the GEBN would develop a website and use social media to ‘provide information and resources about the energy balance approach’ and to disseminate research studies to the public. Finally, the GEBN would look to ‘establish partnerships with global organisations’ such as the American Society for Nutrition, the International Life Sciences Institute, and others that ‘would be sympathetic and supportive of’ Coca-Cola’s initiative. This would be vital for disseminating the GEBN’s message ‘through a variety of channels that reach the public, academic, industry and government audiences’.

## Conclusion

Coca-Cola’s own proposal states: “We propose to establish The Global Energy Balance Network to serve as a focal point for a new collaborative initiative to reduce obesity with strategies that are based on the science of energy balance and on an understanding of both individual and social/cultural behavioral motivation.” One might infer from this a noble intention to establish the GEBN purely in the interest of improving public health. Yet, closer inspection of Coca-Cola’s proposal for establishing the GEBN corroborates long-standing concerns about food and beverage corporations’ involvement in scientific organisations and their similarities with the tobacco industry’s efforts at casting doubt about the links between smoking and cancer.^6^ The comments of those involved in the GEBN also show a less balanced view, as when one of its leading members said that ‘there’s really virtually no compelling evidence’ that fast food and sugary drinks contribute to obesity, despite extensive evidence to the contrary.^3 16–20^

Ultimately, the emails suggest that Coca-Cola proposed and supported the GEBN because it would serve as a ‘weapon’ to ‘change the conversation’ about obesity in its ‘war’ with public health. Despite its close links to Coca-Cola, the GEBN was to be portrayed as an ostensibly ‘honest broker’ while advancing the ‘energy balance’ framework and actively advocating this approach among policy-makers, scientists, health-professionals, journalists and the public. As they note, their intention was to ‘promote practices that are effective in terms of *both policy and profit* (emphasis added)’.

## Footnotes

i The source documents were made available to *JECH* to verify the accuracy of our quotes and characterisations of them, but they are not legally publishable in their original form.
